# Antimicrobial activity of *Paenarthrobacter nicotinovorans*

**DOI:** 10.17912/micropub.biology.000921

**Published:** 2023-08-28

**Authors:** Justyna Kakol, Mainor Vang, Drew Sausen, Tracey Steeno, Angelo Kolokithas

**Affiliations:** 1 Northeast Wisconsin Technical College, Green Bay, Wisconsin, United States

## Abstract

Antibiotic resistance is one of the biggest global challenges of the century. Many pathogens have become resistant to antibiotics due to human misuse and effective ones are in short supply. Discovering potential new antibiotic molecules may help to address the current antibiotic shortage. To this end, the Tiny Earth program, which engages students from around the world to discover potential antibiotic producing microbes from the local soil, was utilized. Students collect, screen, and isolate bacteria from their local communities in hopes of addressing the global crisis of antibiotic resistance. One such microbe, *Paenarthrobacter nicotinovorans* , was isolated and screened for antimicrobial activity.

**Figure 1. Isolation and screening of antimicrobial activity in MV2 bacteria:**
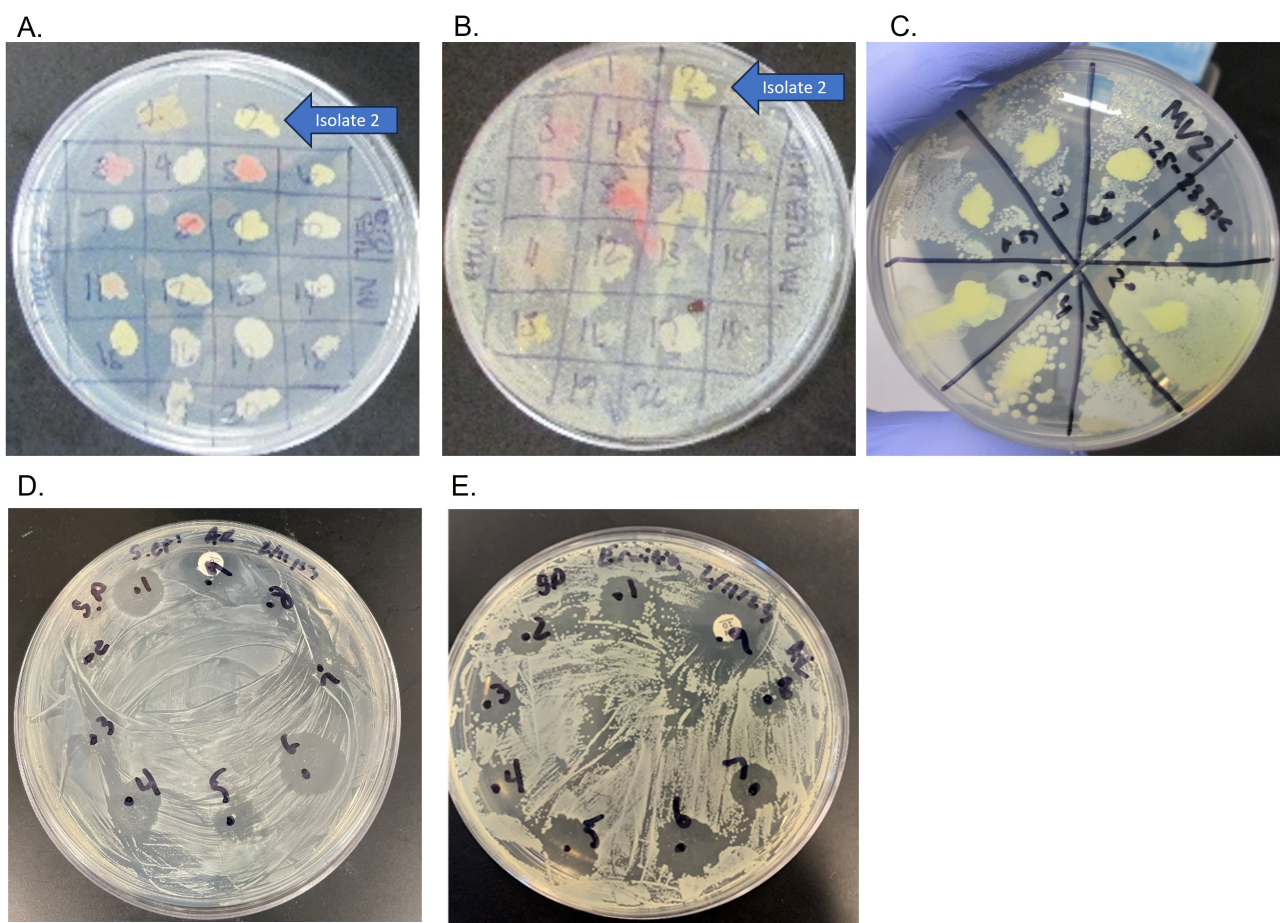
**A.** 20 bacterial colonies were patched onto an LB plate from soil sample. **B.** The 20 isolated bacteria from Figure 1 A. were patched on top of *Erwinia carotovra* and activity (clearing) was checked after 24 hours later. **C.** Sample in spot 2 (MV2) from Figure 1A was patched on top of the following pathogens: (1) *Staphylococcus* *epidermidis* ; (2) *Pseudomonas putida* ; (3) *Enterobacter aerogenes* ; (4) *Mycobacterium smegmatis* ; (5) *Bacillus subtilis* ; (6) *Acinetobacter bacilli;* (7) *Erwinia carotovora* ; (8) *Escherichia coli.* Activity was checked 24 hours later. Testing isolated secondary metabolites against two strains of ESKAPE pathogens: **D.** *Staphylococcus epiermidis* and **E.** *Erwinia carotovora* by using spot-plate method. Numbers correspond to the following isolates. 1. SEC 10 – organic layer of secondary metabolite , 2. BB12- organic layer of secondary metabolite, 3. CM19 - organic layer of secondary metabolite, 4. MV2- organic layer of secondary metabolite with small amount of aqueous layer 5. BS16 - organic layer of secondary metabolite with small amount of aqueous layer 6. MV2 - organic layer of secondary metabolite 7. BS16 – organic layer of secondary metabolite 8. Negative control 9. Positive control.

## Description

Twenty unique colonies from a soil culture were patched onto LB plates for testing ( Figure 1 A ). The 20 bacteria were screened for activity against *Eriwinia* *carotovora* utilizing the plate-patch method and visualized after 24 hours for activity ( Figure 1B ). Bacteria in spots 2 and 17 showed some clearing after 24 hours, and spot 2 (herein referred to as sample MV2) was ultimately selected for further analysis. ESKAPE pathogen safe relatives were plated on to an LB plate, and MV2 was spotted on top ( Figure 1C ). After 24 hours, activity was present against *Staphylococcus epidermidis, Pseudomonas putida, Bacillus subtilis, Acinetobacter bacilli, Erwinia carotovora,* and *Escherichia coli* .

To identify the MV2 bacteria, colony PCR was performed from a single isolated colony from an LB plate using the 16S rRNA primer set to target that gene for analysis. Resultant PCR products were subjected to agarose gel electrophoresis and visualized. The MV2 PCR product was sent for subsequent Sanger sequence analysis at Functional Bioscience Inc. Sequence was analyzed for quality with SnapGene sequence software. The Basic Local Alignment Search Tool (BLAST) tool (National Library of Medicine) was utilized to determine the closest relative to MV2: *Paenarthrobacter nicotinovorans* (accession MT525236.1, 99.73% identity, 100% Query coverage, 0 E value). Phenotypic analysis confirmed the MV2 bacteria was a gram positive rod shaped bacteria that showed starch hydrolysis and positive oxidase activity, which match with *Paenarthrobacter nicotinovorans* (Busse, 2016) .

To isolate the secondary metabolites for antimicrobial activity testing, MV2 was inoculated onto an LB plate and allowed to grow for 48 hours. After incubation, the sample was processed by freezing and organic extraction as described above. The MV2 extracted secondary metabolites were desiccated followed by reconstitution in methanol at a concentration of 10ug/ml. Samples from other students underwent the same processing and were tested alongside MV2. To determine if antimicrobial activity was retained after the extraction protocol, *Staphylococcus epidermid* is and *Erwinia carotovora* were plated onto LB plates. Afterwards, 10ul of each extracted secondary metabolite was added on top of the inoculated media and allowed to dry. Results were visualized after 24 hours ( Figure 1 D and E ). The secondary metabolites of MV2 ( Figure 1 D and E, spot 6) showed activity against *Staphylococcus epidermidis* and *Erwinia carotovora* , respectively. The MV2 sample showed comparable activity to the positive controls and therefore was chosen for further analysis.

At present, the world is facing an antibiotic resistance crisis. As bacterial evolution continues to outpace the development of effective antibiotic therapies, interventions such as those employed by Tiny Earth, are needed. A collaborative effort, combining technical college students across several disciplines and courses, was used to accomplish this study. Though *Paenarthrobacter nicotinovorans* has been described previously, its potential use for antibiotic development has only been tested by the production of silver nanoparticles, and the toxicity of these were not determined (Huq and Akter, 2021) . To the authors knowledge, no other members of the *Paenarthrobacter* genus have been determined to produce antibiotics *.* To this end this bacterium may represent a novel source of new antimicrobial substances, as it had activity against several pathogens ( Figure 1 ). Further studies are needed to elucidate the actual antimicrobial molecules that present this activity as well as further *in vivo* studies. There are several limitations to this study. Here, we provisionally identified MV2 as a close relative of *Paenarthrobacter nicotinovorans* using 16S rRNA sequence. A more through genetic analysis of the whole genome would help further characterize the MV2 sample. The MV2 secondary metabolites were also isolated using basic organic chemistry methods and solvents. More sophisticated isolation methods should be used in the future to isolate specific metabolites. Nonetheless, *Paenarthrobacter nicotinovorans* represents a good candidate for further antimicrobial development.

## Methods

*Bacterial isolation*

*Paenarthrobacter nicotinovorans* (accession MT525236.1, 99.73% identity, 100% Query coverage, 0 E value) was isolated from a soil sample near a park in Green Bay, WI (GPS coordinates: 44.54483779096186, -88.10330001497925) as part of a microbiology course utilizing the *Tiny Earth* protocols (Hurley et al, 2021) . To isolate the bacteria, one gram of soil was diluted in phosphate buffered saline and plated on Luria Broth (LB) plates. Colonies of bacteria were screened for antimicrobial properties against *Erwinia carotovora* and *Staphylococcus epidermidis* . PCR products from isolates showing activity against the screen were sequenced utilizing the 16S rRNA gene primer 357F (Hurley et al, 2021) . Purified bacteria underwent phenotypic testing to verify gram type, cellular morphology, starch, and blood agar activity (Hurley et al, 2021) . The isolate was then preserved in LB/glycerol at -80C and labeled with the identifier MV2.

*PCR and gel electrophoresis*

Colony PCR was performed on a single colony mixed in OneTaq Hot Start Quick-Load 2x Master Mix w/ Standard Buffer to manufacturers specifications along with the 16S rRNA gene primer set (357F-CTCCTACGGGAGGCAGCAG, 1391R- GACGGGCGGTGTGTTCA) in a reaction volume of 25 ul. The cycle parameters were as follows: Stage 1-94°C for 10 minutes. Stage 2-94°C for 30 seconds, 58°C for 30 seconds, 72°C for 1 minute 20 seconds (Stage 2-repeated 25x); Stage 3-72°C for 10 minutes. A colony of *Escherichia coli (* ATCC #11775) was used as a positive control and no colony was used as a negative control. Resultant PCR products were loaded into a 1% agarose gel in TAE buffer and ran at 100V for 1 hour. Visualization was achieved under blue light and DNA bands (1100bp in size) were compared with the Bio DL 200 Standard Ladder (Bulldog Sciences).

*Screening for activity against ESKAPE pathogens*

*Staphylococcus epidermidis (* ATCC #14990), *Pseudomonas putida(* ATCC #12633), *Enterobacter aerogenes (* ATCC #51697), *Mycobacterium smegmatis (Handlesman Lab)* , *Bacillus subtilis (* ATCC #6015), *Acinetobacter bacilli (* ATCC #33305), *Erwinia carotovora (Handlesman Lab)* , *Escherichia coli (* ATCC #11775) were inoculated onto LB agar and then a colony of MV2 was patched onto each tester strain. After 24-48 hours, plates were checked for antimicrobial activity represented by clearing.

*Extraction of secondary metabolites*

MV2 was inoculated as a lawn onto LB agar plates. Forty-eight hours after inoculation the agar was cut into small pieces froze at -80˚C for 30 minutes. Once the agar was frozen, 15 mL of ethyl acetate and 10mL of water was added, and the mixture was shaken gently for 24 hours. When the layers were separated, the top organic layer was transferred into a new vessel and desiccated[1]. Methanol was added to reconstitute the mixture at a concentration of 10mg/ml

*Activity of secondary metabolites*

Onto LB plates, 10 µL of the MV2 secondary metabolites or methanol was added and allowed to dry. Tester strains, *Erwinia carotovora* and *Staphylococcus epidermidis* , were inoculated onto LB plates and spread using spreaders. A positive control, penicillin 10 µg (P10) for gram positive bacteria ( *Staphylococcus epidermidis* ), and tobramycin 10 µg (NN10) for gram negative bacteria ( *Erwinia carotovora* ) were added on top of the tester bacteria. Results were analyzed after 24 hours.
